# Combustion Synthesis of Functionalized Carbonated Boron Nitride Nanoparticles and Their Potential Application in Boron Neutron Capture Therapy

**DOI:** 10.3390/ma17102438

**Published:** 2024-05-18

**Authors:** Stanisław Cudziło, Bożena Szermer-Olearnik, Sławomir Dyjak, Mateusz Gratzke, Kamil Sobczak, Anna Wróblewska, Agnieszka Szczygieł, Jagoda Mierzejewska, Katarzyna Węgierek-Ciura, Andrzej Rapak, Paulina Żeliszewska, Dawid Kozień, Zbigniew Pędzich, Elżbieta Pajtasz-Piasecka

**Affiliations:** 1 Faculty of Advanced Technologies and Chemistry, Military University of Technology, 00-908 Warsaw, Poland; slawomir.dyjak@wat.edu.pl (S.D.); mateusz.gratzke@wat.edu.pl (M.G.); 2 Hirszfeld Institute of Immunology and Experimental Therapy, Polish Academy of Sciences, 53-114 Wrocław, Poland; anna.wroblewska@hirszfeld.pl (A.W.); agnieszka.szczygiel@hirszfeld.pl (A.S.); jagoda.mierzejewska@hirszfeld.pl (J.M.); katarzyna.wegierek@hirszfeld.pl (K.W.-C.); andrzej.rapak@hirszfeld.pl (A.R.); elzbieta.pajtasz-piasecka@hirszfeld.pl (E.P.-P.); 3 Faculty of Chemistry, Biological and Chemical Research Centre, University of Warsaw, 00-927 Warsaw, Poland; ksobczak@cnbc.uw.edu.pl; 4 Jerzy Haber Institute of Catalysis and Surface Chemistry, Polish Academy of Sciences, 30-239 Krakow, Poland; paulina.zeliszewska@ikifp.edu.pl; 5 Department of Ceramics and Refractories, Faculty of Materials Science and Ceramics, AGH University of Krakow, 30-059 Krakow, Poland; kozien@agh.edu.pl (D.K.); pedzich@agh.edu.pl (Z.P.)

**Keywords:** boron nitride, combustion synthesis, boron neutron capture therapy, medical application

## Abstract

In this research, we developed boron-rich nanoparticles that can be used for boron neutron capture therapy as potential carriers for boron delivery to cancerous tissues. Functionalized carbonated boron nitride nanostructures (CBNs) were successfully synthesized in self-propagating combustion waves in mixtures of high-nitrogen explosives and boron compounds. The products’ composition, morphology, and structural features were investigated using Fourier transform infrared spectroscopy, powder X-ray diffraction, low-temperature nitrogen sorption analysis, thermogravimetric analysis, high-resolution scanning electron microscopy, and high-resolution transmission electron microscopy. The extreme conditions prevailing in combustion waves favor the formation of nanosized CBN hollow grains with highly disordered structures that are properly functionalized on the surface and inside the particles. Therefore, they are characterized by high porosity and good dispersibility in water, which are necessary for medical applications. During biological tests, a concentration-dependent effect of the obtained boron nitride preparations on the viability of normal and neoplastic cells was demonstrated. Moreover, the assessment of the degree of binding of fluorescently labeled nanoparticles to selected cells confirmed the relationships between the cell types and the concentration of the preparation at different incubation time points.

## 1. Introduction

Boron nitride (BN) has many polymorphic modifications, such as cubic (*c*-BN), hexagonal (*h*-BN), wurtzite (*w*-BN), rhombohedral (*r*-BN), turbostratic (*t*-BN), explosion (*e*-BN), and amorphous (*a*-BN) phases, but its most stable polymorph at room temperature is the hexagonal form [1,2]. All of these materials exhibit good resistance to corrosion and excellent chemical stability. This material is a prominent candidate for medical applications, including diagnostic purposes and anticancer drug delivery. It can be an attractive boron delivery compound in boron neutron capture therapy (BNCT) because of its high thermal neutron capture cross-section (approx. 3840 barns for ^10^B atoms) [3]. Among these, nanostructured *h*-BN (in the form of nanotubes [4,5,6,7,8,9,10,11], nanosheets [12,13,14,15,16], and nanospheres [17,18,19,20]) has been considered for biological and medical applications. Numerous in vitro and in vivo studies have confirmed that BN materials have better biocompatibility and lower cytotoxicity than their carbon counterparts [21]. Nevertheless, one of the main challenges for integrating all kinds of BN nanostructures into biological systems is their poor dispersibility in water [4,5,6,7,8,9,10,11,12,13,14,15,16,17]. Various methods have been used to improve the water dispersion ability of these materials, such as the surface and edge functionalization of BN particles with hydroxyl and amino groups using strong ultrasonication and ball-milling techniques [22,23,24,25,26,27] or the attachment of OH groups to boron atoms in the central regions of the *h*-BN lattice by solution-phase oxygen radical functionalization [14]. The latter method involves a two-step procedure to initially covalently graft *tert*-butoxy groups to boron atoms and subsequently oxidize the groups to hydroxyl groups [14]. The noncovalent functionalization of BN nanostructures has also been described using alkyl amine, alkyl phosphine, and wrapping with polymers or interactions by other molecules [6,8,9,10,11,15]. However, in many cases, these methods cannot give the desired results, mainly owing to the low modification degrees by the aimed functional groups or guest molecules.

Traditionally, *h*-BN powders with a platelet morphology were prepared by classical high-temperature synthesis methods, including the reaction of boron oxide (B_2_O_3_), boric acid (H_3_BO_3_), or sodium tetraborate (Na_2_B_4_O_7_) with carbon and nitrogen/ammonia or urea/melamine at temperatures up to 2000 °C. Direct nitridation of boron in a nitrogen atmosphere is another well-used route in which the reaction occurs at 1400–1900 °C [28]. Boron nitride nanotubes and nanorods were first prepared via a carbon-free plasma discharge between a BN-packed tungsten rod and a cooled copper electrode [29]. Subsequently, laser ablation, carbothermal reduction in B_2_O_3_ and B_4_C, and other methods have been developed to synthesize BN nanotubes and nanowires [30,31]. At present, the catalytic chemical vapor deposition (CVD) method is one of the most popular techniques for preparing BN nanotubes. Commonly, diborane (B_2_H_6_), ammonia borane (BH_3_NH_3_), trimethyl borate (C_3_H_9_BO_3_), iron boride (FeB), and H_3_BO_3_ are used as precursors [2,31]. Spherical BN submicronic particles were first synthesized through aerosol-assisted vapor synthesis (AAVS) [18,19,20]. A solution of trimethyl borate in methanol was applied as an aerosol precursor to produce, by reaction with ammonia, BN particles with a high production rate, low oxygen and carbon contents, and an average diameter of approximately 30 nm [19]. A simple method was reported in 2014 for synthesizing *h*-BN particles with similar sizes and morphologies using a direct reaction of boric acid and ammonium chloride in the presence of copper oxide at 950 °C under nitrogen [17].

BNCT is a targeted chemo-radiotherapy dedicated to treating hard-to-reach tumors based on nuclear capture and fission after irradiating nonradioactive isotope boron-10 (^10^B) with low thermal neutrons. This therapy utilizes boronated agents to selectively deliver boron to tumor cells [32]. BNCT aims to make personalized tumor radio treatment possible, limiting its therapeutic effect solely to the growing tumor. Therefore, there is a need to design and characterize new selective nanocarriers without systemic toxicity. These potential compounds should be able to accumulate ^10^B at sufficiently high concentrations inside tumor cells. Despite the use of two compounds (sodium boronocaptate (BSH), L-p-boronophenylalanine (L-BPA)) in BNCT, new boron agents are required to treat severe and inoperable malignant tumors [3]. Therefore, we proposed the use of newly synthesized carbonated boron nitride nanoparticles as potential boron delivery carriers to cancerous tissues. A high thermal neutron capture cross-section of boron-10-enriched BN can be suitable for the absorption and interaction of neutrons required in BNCT [33]. Previous research on selected cancer cell lines, HeLa and MCF-7 human adenocarcinoma, demonstrated the potential of boron nitride as a boron donor in boron neutron capture therapy [3].

In this work, we present a new method to synthesize nanometer-sized particles of carbonated boron nitride (CBN) phases in combustion waves propagating in solid mixtures of oxygen-free, low-carbon, and nitrogen-rich explosives (sources of elemental nitrogen and carbon) and boron compounds (sources of elemental boron). In the combustion wave and next in the expanding combustion products, boron reacts with nitrogen, carbon, and/or nitrogen-hydrogen species, forming particles of CBN composites. This is a pure self-sustaining combustion synthesis of CBN solid solutions from elements in their reactive “in statu nascendi” forms. Combustion synthesis (CS) processes proceed at high temperatures along with extremely fast heating and cooling rates of reactants [34]. Thus, they provide ideal conditions for the formation of nanometer-sized particles of BN or CBN phases. Moreover, the resulting BN/CBN nanoparticles contain covalently bound amino and imino groups, as the reactions take place in a mixture composed of mainly nitrogen-hydrogen species formed under highly nonequilibrium conditions (rapidly changing temperature) as well as hydroxyl groups introduced into the structures during postsynthesis treatment—cryogenic exfoliation and ultrasonic fracturing in a water suspension. These functional groups have desirable hydrophilic properties and can be used to functionalize further CBN particles, which is essential for medical applications, such as boron neutron capture therapy. A preliminary assessment of the biological activity of the obtained nanoparticles demonstrated the ability of various cell types to interact with the newly synthesized compounds. Additionally, the range of concentrations at which the nanometer-sized particles showed toxicity in in vitro tests was determined for each cell line. In summary, after further surface functionalization toward the tumor environment, the obtained nanoparticles may become potential boron carriers for use in BNCT.

## 2. Materials and Methods

### 2.1. Synthesis and Characterization of Boron Nitride Nanoparticles

Bis(triaminogunidinium) azotetrazolate (TAGAZ, C_4_H_18_N_22_) was synthesized at the Military University of Technology according to a previously published procedure [35]. The purity of the reagent was greater than 99%. Ammonia borane (BH_3_NH_3_) and sodium borohydride (NaBH_4_) were purchased from ABCR GmbH and ACROS Organics, respectively, and were used as received. The solvents, *h*-BN micrometer-sized powder, boric acid, and other chemicals used were of chemical purity or purer and were purchased from Sigma Aldrich (St. Louis, MO, USA).

FTIR measurements were recorded using a Nicolet iS5 instrument with a diamond ATR iD7 accessory. X-ray powder diffraction patterns were obtained on a Rigaku Ultima IV X-ray diffractometer (Rigaku, Tokyo, Japan) with a cobalt lamp (λ = 0.179 nm) using cross-beam optics in parallel beam mode. The voltage and current were 40 kV and 40 mA, respectively. Combined TGA-DTA thermal analyses were performed using an STA 449 F5 Jupiter (NETZSCH-Gerätebau GmbH, Selb, Germany) apparatus. The samples were heated from 30 to 1200 °C at 10 °C/min in nitrogen or synthetic air flowing at 50 mL/min. The specific surface area (calculated using the BET model) was determined from the nitrogen adsorption–desorption isotherm measured at −196 °C on an autosorb iQ analyzer (Quantachrome Instruments, Boynton Beach, FL, USA).

The morphology and size distribution of the CBN samples were determined by a Ultra Plus field emission scanning electron microscope (SEM) (Carl Zeiss AG, Oberkochen, Germany) operated at 30 kV. Transmission electron microscopy (TEM, 80 kV) was conducted using a JEM-1200 EX instrument (JEOL, Tokyo, Japan). High-resolution TEM investigations were performed on an Talos F200X transmission microscope (Thermo Fisher Scientific, Waltham, MA, USA) at 200 kV. The morphology and chemical composition were determined in TEM and STEM modes using high-angle annular dark-field imaging (HAADF). An energy-dispersive X-ray spectroscopy (Super-EDS by FEI, Hillsboro, OR, USA) detector was used to map the element distribution. The powdered BN-14 and BN-17 samples were suspended in ethanol (99.8% purity). The specimens for the TEM observations were prepared by dropping colloid on a carbon film supported on a 300-mesh copper grid. In one case, the test sample was prepared using an aqueous suspension that was obtained by first cryogenic pretreatment and then long-term ultrasonic fracturing and exfoliation of BN-17 powder. X-ray photoelectron spectroscopy (XPS) measurements were performed using a PHl 5000 VersaProbe^TM^—Scanning ESCA Microprobe (ULVAC-PHI, Chigasaki, Japan) instrument. XPS spectra were recorded using monochromatic Al–Kα radiation (hν = 1486.6 eV) from an X-ray source operating at a 100 µm spot size, 25 W, and 15 kV. High-resolution (HR) XPS spectra were collected with an analyzer pass energy of 23.5 eV and an energy step size of 0.1 eV. Casa XPS software (v.2.3.19, Casa Software Ltd., Wilmslow, UK) was used to evaluate the XPS data. The binding energy scale was referenced to the C 1s peak with BE = 284.8 eV. The spectrometer’s function was used to quantify the PHI Multipak sensitivity factors and determine transmission.

A Zetasizer Nano ZS instrument from Malvern (Malvern Instruments, Malvern, UK) was used to measure the diffusion coefficient by dynamic light scattering (DLS) and electrophoretic mobility by laser Doppler velocimetry (LDV). The hydrodynamic diameters were calculated using the Stokes–Einstein relationship, and the zeta potential was calculated using the Smoluchowski model.

### 2.2. Cell Cultures

MC38 in vivo-grown murine colon carcinoma cells from the Tumor Bank of the TNO Radiobiology Institute (Rijswijk, The Netherlands) were adapted to in vitro conditions as described by Pajtasz-Piasecka et al. [36]. MC38/0 (hereafter referred to as MC38) cells were maintained in RPMI 1640 medium (Gibco, Thermo Fisher Scientific, Inc., Waltham, MA, USA) supplemented with 100 U/mL penicillin, 100 mg/mL streptomycin, 1 mM sodium pyruvate, 0.05 mM 2-mercaptoethanol, and 5% fetal bovine serum (FBS) (all from Sigma–Aldrich, St. Louis, MO, USA). THP-1 human monocytic cells obtained from the ATCC ( Manassas, VA, USA) (TIB-202^TM^) were cultured in RPMI 1640 medium (Gibco, Thermo Fisher Scientific, Inc., Waltham, MA USA) supplemented with 100 U/mL penicillin, 100 mg/mL streptomycin, 1 mM sodium pyruvate, 0.05 mM 2 mercaptoethanol, and 10% FBS (all from Sigma–Aldrich). HT-29 human colon adenocarcinoma cells were obtained from ATCC (Manassas, VA, USA) (HTB-38^TM^), and RAW 264.7 mouse macrophages were obtained from ATCC (Manassas, VA, USA) (TIB-71^TM^) and maintained in Dulbecco’s Modified Eagle’s Medium (DMEM; ATCC, Manassas, VA, USA) supplemented with 100 U/mL penicillin, 100 mg/mL streptomycin, and 10% FBS (all from Sigma–Aldrich, St. Louis, MO, USA). All cell cultures were maintained at 37 °C, 95% humidity, and 5% CO_2_ at the Hirszfeld Institute of Immunology and Experimental Therapy, Polish Academy of Sciences, Wrocław, Poland.

### 2.3. MTT Cell Proliferation Assay

MC38, RAW 264.7, and HT-29 cells (5 × 10^3^ cells/100 µL/well) and THP-1 cells (1 × 10^4^ cells/100 µL/well) were seeded in 96-well plates (Corning Costar, Corning, NY, USA). After 24 h, CBN preparations (BN-14, BN-17) were diluted to specific concentrations (range 0.1–400 µg/mL) and added to the plates (100 µL/well). The cells were incubated with compounds for the next 72 h. Afterwards, MTT dye (3-(4,5-dimethylthiazol-2-yl)-2,5-diphenyltetrazolium bromide; 5 mg/mL) (Sigma–Aldrich, St. Louis, MO, USA) was added to a final concentration of 0.55 mg/mL (25 µL/well) and incubated for 4 h, followed by the addition of 75 µL/well of lysis buffer (N,N-dimethylmethanamide, sodium dodecyl sulfate, and Milli-Q water) and incubation overnight at 37 °C. Finally, the absorbance at 570 nm was measured using a Thermo Labsystems Multiskan RC microplate reader (Thermo Fisher Scientific, Inc., Waltham, MA, USA) with Genesis Lite 3.05 software (Thermo Life Sciences, Waltham, MA, USA). The 50% inhibition of cell proliferation (IC_50_) values were calculated using GraphPad Prism 9 software (GraphPad Prism Software version 9.0.0, La Jolla, CA, USA).

### 2.4. Preparation of FITC-Labeled BN-17

A CBN sample was suspended in 10 mM borate buffer (pH 8.0) (Sigma–Aldrich St. Louis, MO, USA). Next, 100 µL of fluorescein isothiocyanate (FITC; 1 mg/mL) (Sigma–Aldrich St. Louis, MO, USA) was added, and the suspension was incubated overnight at 4 °C. The preparation was centrifuged for 10 min at 20,000× *g* and washed three times with water. Finally, the labeled boron nitride was suspended in 1 mL of water.

### 2.5. Flow Cytometry Analysis

RAW 264.7 cells (0.05 × 10^6^/0.5 mL/well) and THP-1, MC38, and HT-29 cells (0.1 × 10^6^/0.5 mL/well) were seeded in 48-well plates (Corning Costar, Corning, NY, USA). The next day, FITC-labeled BN-17 at concentrations of 1, 50, and 100 µg/mL was added. After 4 and 24 h of incubation with the compound, the cells were collected and suspended in PBS supplemented with 2.5% FBS (Biowest, Nuaillé, France). The mean fluorescence intensity (MFI) as well as changes in the size and granularity of cells were analyzed based on forward scatter (FSC) versus side scatter (SSC) using a FACS Fortessa instrument with Diva software version 8.0 (Becton Dickinson). DAPI dye (Thermo Fisher Scientific, Inc., Waltham, MA, USA) was added to each tube to eliminate dead cells. Histograms and density dot plots were prepared using NovoExpress 1.3.0 software (Agilent Technologies, Inc., Santa Clara, CA, USA).

### 2.6. Fluorescence Microscopy

RAW 264.7 cells (0.5 × 10^4^ cells/well) were seeded in a black 96-well plate (Corning Costar, Corning, NY, USA) and incubated with 100 µg/mL FITC-labeled BN-17 for 24 h. After this time, the cells were fixed with 4% paraformaldehyde (Sigma–Aldrich, St. Louis, MO, USA) for 20 min. Then, it was rinsed 3 times with phosphate-buffered saline (PBS) and permeabilized with 0.25% Triton X-100 (Sigma–Aldrich, St. Louis, MO, USA) solution for 15 min. After this time, the cells were rinsed again with PBS and stained with DAPI dye (Thermo Fisher Scientific Inc., Waltham, MA, USA) at a concentration of 0.1 µg/mL for 15 min. The cells were visualized using an Olympus IX81 inverted fluorescence microscope (Olympus, Tokyo, Japan).

### 2.7. Statistical Analysis

All the data were analyzed using GraphPad Prism 9 software (version 9.0.0, La Jolla, CA, USA). The significant differences were calculated using the unpaired *t*-test for comparisons between two groups. In other cases, two-way ANOVA followed by Tukey’s multiple comparison post hoc test was performed. The type of statistical analysis used is described in the captions under the figures. All statistically significant differences are presented in the graphs; otherwise, the differences were not significant.

## 3. Results

### 3.1. Synthesis and Purification of CBNs

To obtain pure BN nanoparticles during the combustion process, it is necessary to use explosive compounds without oxygen and carbon in the molecules. Oxygen should be excluded to avoid the competing processes of boron oxidation during synthesis. Unfortunately, there are no explosive compounds containing nitrogen or nitrogen and hydrogen exclusively (excluding ammonium azide (NH_4_N_3_), which is an unacceptably sensitive explosive). Therefore, we used bis(triaminogunidinium) azotetrazolate (TAGAZ, C_4_H_18_N_22_, 82.3% N). It was mixed with sodium borohydride (NaBH_4_) or ammonia borane (BH_3_NH_3_) and burned in a reactor. The syntheses were performed under a nitrogen atmosphere to further facilitate the reaction.

In a typical experimental procedure, a 10 g sample of a homogeneous powdery mixture containing 60 ÷ 80% TAGAZ and 40 ÷ 20% NaBH_4_ or BH_3_NH_3_ was poured into a steel crucible and placed in a 2 L stainless steel reactor. The reactor was purged and filled with nitrogen at different pressures. The reaction was initiated with an electrically heated resistance wire immersed in the powdered sample. After cooling and depressurizing the reactor, the obtained white to light-gray solid was collected and washed to remove byproducts. The first-stage products were dried and then annealed at 1000 °C in an argon atmosphere to improve the crystallinity and remove volatile impurities. To fragment the particles of the CBN powders thus obtained, the recently proposed process of cryogenic-mediated exfoliation followed by ultrasonication fracturing of layered materials was applied [37]. The method relies on liquid nitrogen pretreatment of bulk powder, which is then thermally shocked by dispersing it in room-temperature water. The obtained suspension of CBN particles was exposed to an ultrasonication bath for an extended period to induce full liquid-phase exfoliation.

The sample referred to as BN-14 is a product of the reaction in the TAGAZ/NaBH_4_ = 70/30 mixture carried out in a nitrogen atmosphere at atmospheric pressure. In contrast, the BN-17 sample was obtained by burning a mixture of TAGAZ/BH_3_NH_3_ = 70/30 under the same conditions. The crude products were washed successively with distilled water, diluted hydrochloric acid, deionized water, and ethanol several times, heated in a stream of argon from 30 °C to 1000 °C at a heating rate of 10 °C/min, and subsequently kept at this temperature for half an hour. After cooling to room temperature, they were soaked in liquid nitrogen for one hour. The liquid nitrogen suspensions were dispersed into room-temperature water, and the obtained CBN suspension in water was kept in an ultrasonic bath for 10 h. Next, the mixture was left for 24 h to allow all insoluble materials and aggregates to precipitate. The translucent milky-white solutions were decanted from the sediments to obtain stable aqueous suspensions of BN-14 and BN-17 with concentrations of approximately 0.5 mg/mL and 2.0 mg/mL, respectively.

### 3.2. Characterization of CBN Samples

**Figure 1:**
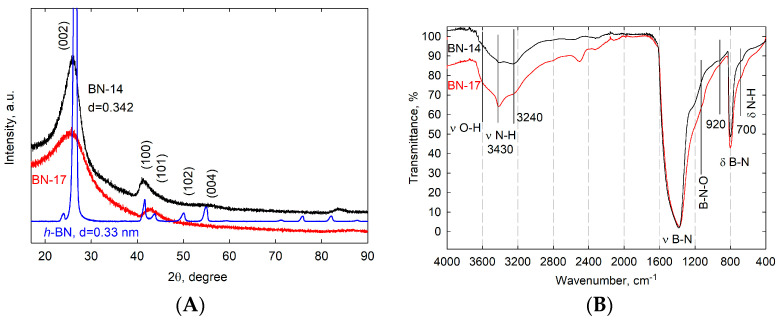
(**A**)—XRD patterns of well-crystallized *h*-BN and the BN-14 and BN-17 samples. (**B**)—FTIR spectra of the BN-14 and BN-17 samples.

Figure 1A,B show powder X-ray diffraction patterns and Fourier transform infrared (FTIR) spectra of the combustion synthesis (CS) products obtained using NaBH_4_ (BN-14) and BH_3_NH_3_ (BN-17) as boron carriers, the processing of which ended with annealing at 1000 °C in an argon atmosphere.

The hexagonal phase of well-crystallized *h*-BN is characterized by peaks at 26.8°, 41.7°, 43.9°, 50.2°, and 55.2°, which correspond to the (002) (d_002_ = 0.33 nm), (100), (101), (102), and (004) planes, respectively (JCPDS card No. 34-0421) (Figure 1A). In contrast, the BN-14 and BN-17 XRD patterns reveal diffuse diffraction peaks at 2θ values of approx. 26.2° and 42° that indicate the existence of B-N-containing phases in the tested materials, but the peak position offset (as compared to that of crystalline *h*-BN) and broadness, as well as the absence of other diffraction lines characteristic of *h*-BN, can be attributed to the amorphization of the BN structure (formation of *a*-BN and *t*-BN areas), nanometer-scale effects, and, above all, to impurity incorporation—most likely in the form of carbon substitution of boron and nitrogen atoms and functionalization of the formed CBN structures with hydroxyl and amino groups. The extremely fast heating/cooling rate associated with the very short residence time at the highest temperature involved in the combustion process inherently leads to the poor crystallization of CBN composites and the existence of impurities, i.e., other elements permanently connected with the CBN matrix. Despite this, the presence of weak (002) and (100 fused with 101) diffraction peaks proves that hexagonal in-plane ordering still occurs [25]. A slight shift of the (002) peak from 2θ = 26.8° (*h*-BN) to 26.2° (CBN) indicates an increase in layer interspacing (d_002_ = 0.342 nm).

FTIR analyses further confirmed *h*-CBN phase formation and the presence of impurities (Figure 1B). For *h*-BN, two dominant IR absorption peaks at ~1380 and ~800 cm^−1^ are assigned to in-plane B-N stretching and out-of-plane B-N-B bending vibrations, respectively. These results indicate the backbone role of the B-N networks for these combustion synthesis products. In addition to the typical B-N vibrations, other absorption bands are associated with C, H, and O bonding. The latter may be due to the hydrolysis of B-H bonds remaining in crude CS products (as well as boron atoms in defective BN layers) during their purification and sonication in water.

The high-frequency region of the spectra exhibits a broad absorption band (between 3700 cm^−1^ and 2800 cm^−1^) centered at ~3430 cm^−1^ and with a shoulder at ~3240 cm^−1^, which can be ascribed to the asymmetric and symmetric stretching modes of B-NH_2_ species [14,19,38]. The shoulder at 3600 cm^−1^ derives from the O-H stretching vibrations peculiar to surface species such as sp^3^-hybridized N_3_B(OH) units [13]. In the low-frequency region of the IR spectra, the CS products exhibit peaks at 1150, 920, and 700 cm^−1^. The bands at 1150 and 920 cm^−1^ could be attributed to the pseudotetrahedral geometry of the boron oxynitride (B-N-O) vibrations. The deformation of N-H bending is characterized by a shoulder at approximately 700 cm^−1^ [19].

XPS analysis was performed to obtain deeper insight into the chemical state and elemental composition of the BN-14 and BN-17 samples. The wide-survey XPS spectrum indicates that both contain boron, nitrogen, oxygen, and carbon (Figure S1, Supporting Information). The chemical formulas (BN-14: BN_0.71_C_0.10_O_0.09_ and BN-17: BN_0.85_C_0.51_O_0.52_) derived from the XPS surveys confirm the extra high carbon and oxygen contents of BN-17 (approx. 18 at%). In addition, the atomic B/N ratios are much greater than 1, especially for the BN-14 sample (insert in Figure S1). It should be noted that the real carbon and oxygen contents in the samples may be different than the values given in the insert because XPS probes only the top few nanometers of a material’s surface.

**Figure 2:**
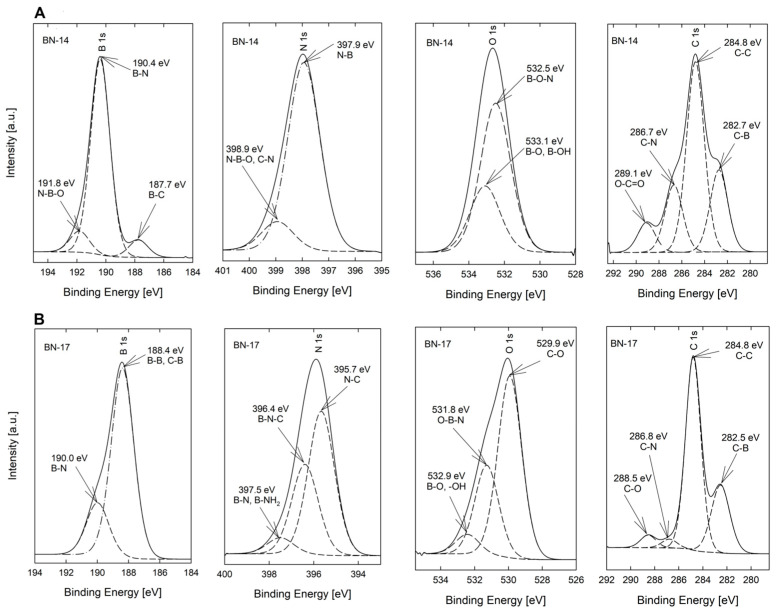
High-resolution XPS spectra of B1s, N1s, C1s, and O1s for BN-14 (**A**) and BN-17 (**B**).

The deconvolutions of the B1s, N1s, O1s, and C1s edges are shown in Figure 2. The main peak of B1s at 190.4 eV in the spectrum of BN-14 is attributed to B atoms surrounded by N atoms in the *h*-BN planes [39]. Correspondingly, the more intense component of the N1s spectrum at 397.9 eV is due to N atoms bound only to B atoms, and the other small peak at 398.9 eV can be ascribed to boron oxynitrides as well as to C-N bonding in the sp^3^ tetrahedral form [39]. Both the B1s and N1s spectra indicate that the main configuration for B and N atoms in BN-14 is related to B-N bonding, implying boron nitride formation. The subpeaks at 191.8 eV and 187.7 eV of B1s are attributed to boron atoms in a mixed oxygen/nitrogen environment and boron atoms covalently bonded to carbon atoms, respectively. Oxygen is more electronegative than nitrogen and shifts the binding energy upward, while less electronegative carbon lowers the energy. The O1s subpeak centered at 533.1 eV corresponds to the binding energy of O atoms in B_2_O_3_ (533.2 eV [40]), whereas the second component at 532.5 eV indicates that the O atoms not only bond with the B atoms but also interact with the N atoms, which causes the observed redshift of the binding energy. The main peak of C1s at 284.8 eV is close to the value for graphite (284.5 eV [39]). The component peaking at 282.7 eV can be associated with the presence of C-B bonds, and the two other peaks are assigned to the N-C sp^3^ hybridized state (286.7 eV) [39] and C(O)O (289.1 eV) bonds [41]. It is worth noting that the C atoms are mainly connected to each other, which suggests the presence of separated BN and C phases in BN-14.

The B1s spectrum of BN-17 consists of a main peak at 188.4 eV with a shoulder at 190.0 eV. The former peak should be attributed to B-B bonds in B_4_C and boron atoms bound to carbon atoms in the carbonated BN network [42]. The last peak can be assigned to boron atoms surrounded mainly by nitrogen atoms in the BN planes. Nevertheless, it is shifted from 190.4 eV to 190.0 eV because the boron atoms are in the mixed nitrogen/carbon environment of the CBN composite. The O1s spectrum indicates that approximately 60% of the oxygen atoms on the surface of the BN-17 particles are connected to carbon atoms in the form of carbonyl and hydroxyl functional groups (529.9 eV). The remainder are bound as boron oxynitrides (531.8 eV), oxides, or hydroxides (532.9 eV) [40]. The C1s spectrum of BN-17 can be decomposed into four distinguishable components. The main peak centered at 284.8 eV is attributed to C-C bonds in graphite. The small peaks with the highest binding energies correspond to C–O (288.5 eV) and C–N (286.8 eV) bonds. The low-energy peak (282.5 eV) is due to C-B bonds in the CB_2_N_3_ or C_2_BN_3_ rings. The corresponding signals of N and B atoms for C-N or C-B bonds are also observed as N1s and B1s subpeaks, located at 396.4 eV, 395.7 eV, and 188.4 eV, respectively [39,41]. As with BN-14, the carbon atoms in BN-17 are directly linked to each other with a 40–50% fraction of existing C-N or C-B bonds, which suggests a highly heterogeneous structure with phase-separated boron nitride, graphite, boron carbide, and carbon nitride fragments. On the other hand, XPS is not very sensitive to differences in neighboring atoms when the same structure is involved [42]. Therefore, the presence of boron-substituted graphite phases and carbon-substituted BN phases cannot be ruled out since carbon and boron have the same trigonal network, and the differences in electronegativity between these elements are minor. For this reason, we believe (like the authors of [42]) that the B1s peak at 188.4 eV (BN-17) and the C1s peak at 284.8 eV (BN-14 and BN-17) should be assigned to the in-plane boron atoms in carbonated boron nitride layers and to C-C bonds in boronated graphite domains.

**Figure 3:**
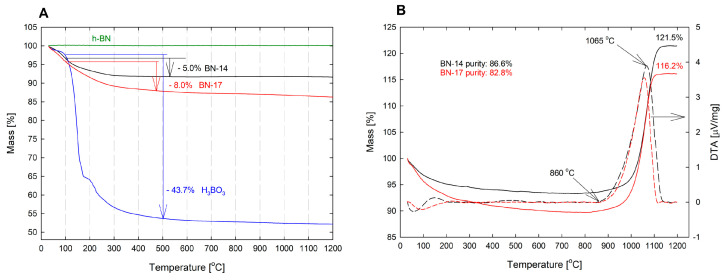
(**A**)—TGA traces recorded in nitrogen for commercial *h*-BN (stable profile up to 1200 °C), the tested samples, and H_3_BO_3_. (**B**)—TGA and DTA curves of the tested samples recorded in the oxidizing atmosphere.

TGA-DTA combined analyses of BN-14 and BN-17 were performed in a nitrogen or synthetic air atmosphere to quantify the number of hydroxyl groups grafted to the BN/BNC layers and to estimate the sample purity by measuring their mass increase due to oxidation. Representative TGA traces recorded in a nitrogen atmosphere for the BN-14 and BN-17 samples and for commercial *h*-BN micrometer-sized powder and analytical grade boric acid, as well as TGA and DTA curves of the tested samples recorded in an oxidizing atmosphere, are shown in Figure 3A,B. Boric acid was chosen as a model compound in which the hydroxyl groups are covalently bonded to boron. When heated, H_3_BO_3_ dehydrates in the temperature range from 100 to 500 °C. In the same temperature range, the tested CBN materials lose water; therefore, their boron atoms are also hydroxylated.

Figure 3A shows that in the temperature range of 100–500 °C, the mass losses of BN-14 and BN-17 are ~5.0% and ~8.0%, respectively, while the mass loss of the reference *h*-BN sample remains constant up to 1200 °C. The dehydration of boric acid starts at approximately 100 °C and ends at approximately 500 °C. The mass of the sample decreases by ~43.7%, corresponding to the following reaction: 2 H_3_BO_3_ → B_2_O_3_ + 3 H_2_O. This indicates that the observed significant mass reduction in BN-14 and BN-17 above 100 °C can be attributed to the loss of hydroxyl groups covalently bound to boron atoms. Taking the mass of the hydroxyl group into account and assuming that OH groups are bound only to boron atoms, the measured mass loss of 5% and 8% yields a functionalization of 7.9 at% and 13.5 at% of the boron atoms in the BN/BNC lattice for BN-14 and BN-17, respectively. This implies that samples BN-14 and BN-17 must be functionalized with hydroxyl groups on the surface and inside the particles, i.e., the OH groups are located between the BN and BNC layers.

When the BN samples are heated in an oxygen-containing atmosphere, the nitrogen atoms are exchanged for heavier oxygen atoms. Therefore, the purity of the test sample can be estimated by measuring its weight gain (Figure 3B). Figure 3B shows that under the experimental conditions, both samples began to oxidize (to gain mass) at approximately 860 °C. The reactions were completed at approximately 1150 °C. At this temperature, the mass of the BN-14 sample increased by 21.5% and that of the BN-17 sample increased by 16.2%. After the complete oxidation of pure BN according to the reaction of 2 BN + 1.5 O_2_ = B_2_O_3_ + N_2_, the mass increase should be 40.25%. Thus, BN-14 contains 86.6% BN, while the purity of BN-17 is ~82.8%.

**Figure 4:**
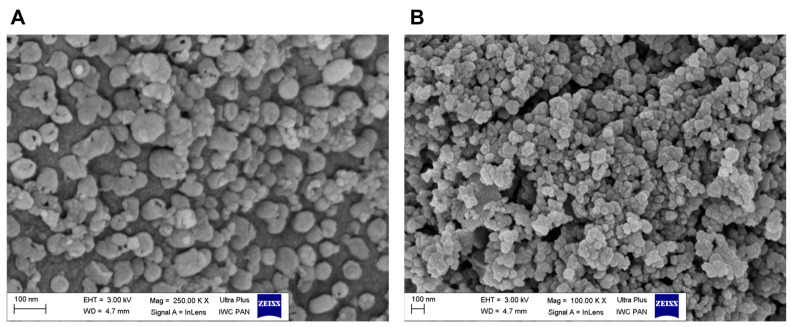
(**A**)—HR SEM image of BN-14 particles, (**B**)—HR SEM image of BN-17 particles.

Figure 4A,B show typical SEM images of the BN-14 and BN-17 powders. In both cases, CBN particles have a spheroidal shape and nonsmooth surface, and their size does not exceed 100 nm. The morphology of the BN-17 powder is more uniform in shape and particle size, but the particles are agglomerated, whereas the BN-14 particles are relatively well dispersed. Some have open pores on the surface, indicating that they are hollow.

**Figure 5:**
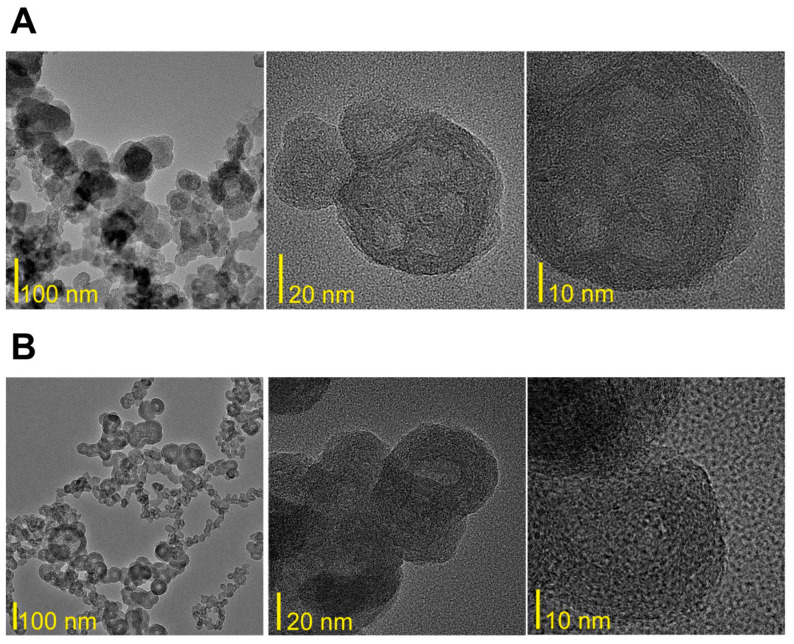
(**A**)—Low- and high-magnification HRTEM images of the powdery BN-14 sample. (**B**)—Low- and high-magnification HRTEM images of the powdery BN-17 sample.

In-depth characterization of the morphology, as well as the textural features and chemical composition of the synthesized samples, was performed using HRTEM, first, to ensure that the procedure produced highly hydroxylated, hexagonal in-plane local structures containing boron, carbon, and nitrogen (carbon-substituted BN) and, second, to ensure that the layered structure of the particles remained intact. Figure 5A,B present representative HRTEM images of the powdery BN-14 and BN-17 samples, the processing of which ended with annealing at 1000 °C. The low-magnification TEM bright field images of both powders show that they are composed of aggregated spheroidal nanoparticles. Nevertheless, their easy dispersion in the solvent proved that the level of aggregation was low, especially in the case of BN-17. The local particle sizes are less than 100 nm, which aligns with the results of the SEM observations (see Figure 4).

HRTEM observations confirmed the lack of long-range order and the heterogeneity of the crystal structure. The bright center and dark edges indicate that the particles of BN-14 and BN-17 have a hollow core within their interior. Round core-shell grains are built of several dozen concentric layers with high tortuosity in their stacking sequence, as commonly observed in precursor-derived BN [18,38].

High-angle annular dark-field (HAADF) imaging was conducted to track the elemental distribution within the particles of BN-14 and BN-17. HAADF images (Figure S2, Supporting Information) indicate the presence and uniform distribution of boron, nitrogen, carbon, and oxygen atoms over the entire area of the BN-14 and BN-17 samples. The uniform distribution of carbon atoms shows that they do not form a separate phase but are built into the BN layers, substituting for boron and nitrogen atoms. Additionally, considering that the XPS measurements showed a dominant share of carbon-to-carbon bonds, the C atoms must be adjacent in the trigonal in-plane local CBN structures. The oxygen atoms are also uniformly distributed; thus, the formation of nanometer-sized O-rich particles such as boron oxide or boric acid in the samples can be excluded.

**Figure 6:**
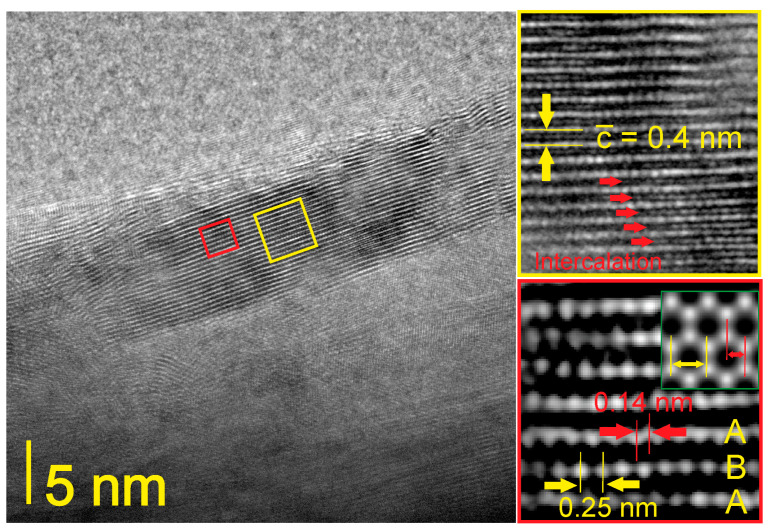
Lattice fringe image of intercalated CBN planes in the BN-17 sample from a water suspension.

Detailed XPS and TGA-DTA analyses demonstrated the chemical bonding of OH groups on the CBN surfaces. Additionally, the layered hexagonal arrangement of B, N, and C atoms and the localization of hydroxyl and probably also amino groups between the layers were further verified by HRTEM (Figure 6). The specimen for HRTEM observations was prepared from a water suspension of BN-17, which was finally examined in the biological part of the work. These suspensions were obtained by soaking BN-17 powder in liquid nitrogen, pouring the nitrogen suspension into room-temperature water, and keeping the resulting water suspension in an ultrasonic bath for 10 h. We assumed that cryo-mediated exfoliation and ultrasonic fracturing resulted in a change in the morphology of some particles from spherical to plate-like and their interlayer functionalization with hydroxyl groups, which in turn caused an increase in the interlayer distance in some domains. As seen in the upper inset (yellow-framed area), layers are most likely separated by hydroxyl and amino groups because the interplanar spacing (002) is approximately 0.4 nm, while in a pure, well-crystallized *h*-BN, this parameter equals 0.333 nm [43]. Moreover, the spaces between the CBN layers seem to be filled with guest molecules.

The lower inset in Figure 6 is a reconstructed reverse fast Fourier transform image from the red-framed area. This confirms the hexagonal atom configuration in the layers. The spaces between the BN/CBN layers are empty in this fragment. The center distance between adjacent hexagonal rings is ~0.25 nm, and the bond length in the ring is ~0.14 nm, which corresponds to the atom separations both in *h*-BN (0.144 nm) and in graphite (0.142 nm) [43]. This implies that the BN-17 sample does not have a homogenous B-C-N composition but contains adjacent nanometer-sized, nonfunctionalized B/N-rich domains and regions of carbonated BN, the layers of which are intercalated with chemically bonded hydroxyl and amino groups.

EDX analyses of the samples were also performed to corroborate the XPS results. The EDX spectra, taken from the same area as the HAADF maps, clearly demonstrate the presence of large amounts of boron carbon, nitrogen, and oxygen (Figure S3, Supporting Information). The Cu peaks arise from the supporting copper grid used. A small amount of silicon (0.13–0.21 at%) was probably introduced into the samples during their purification, as this process was carried out in a glass apparatus, and the solutions used were alkaline. The samples for TEM measurements were deposited on a carbon film; therefore, the intensity of the C peaks is very high.

Finally, to study the porosity of the CBN materials, N_2_ sorption analyses at −196 °C were conducted (Figure S4, Supporting Information). The isotherms exhibited type IV with H1 hysteresis loops at relative pressures between 0.45 and 0.98, suggesting the formation of mesoporous carbonated boron nitrides (according to the IUPAC classification [44]). The specific surface area (SSA) of the tested samples was greater than 200 m^2^/g, so their drug loading and release capability should be good. The slightly higher SSA of BN-14 may result from the larger quantity of mesopores in BN-14 than in BN-17 because the SEM observations showed that some BN-14 particles appeared as hollow spheres.

### 3.3. Physicochemical Characteristics of BN-14 and BN-17 Nanoparticles

**Figure 7:**
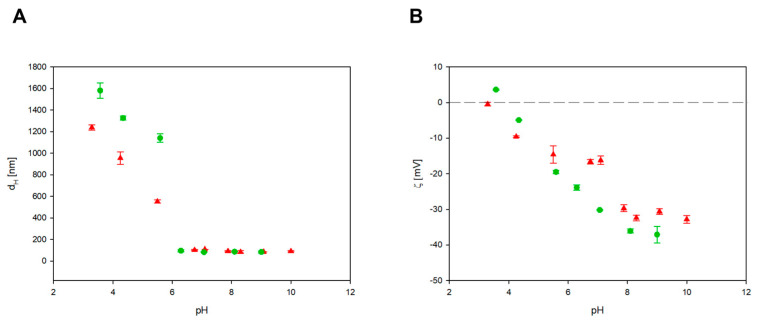
Dependence of the hydrodynamic diameter (**A**) and zeta potential (**B**) of BN-14 (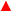) and BN-17 (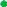) on pH at a NaCl concentration of 0.0001 M. The dashed line shows the zero value of the zeta potential.

DLS diffusion coefficient measurements revealed that the CBN particle suspension remained stable at pH values above 6. As shown in Figure 7A, the hydrodynamic diameters of BN-14 and BN-17 were 90 ± 10 nm and 85 ± 9 nm, respectively, for pH values greater than 6. In comparison, for pH values lower than 6, the hydrodynamic diameter increases dramatically, indicating particle aggregation.

The zeta potential of the CBN particle suspension decreased with increasing pH, and at a pH of approximately 10, the zeta potentials were −33 ± 2 mV and −37 ± 3 mV for BN-14 and BN-17, respectively. For convenience, these values are graphically presented in Figure 7B for a broad range of pH values. The isoelectric point of CBN (defined as the pH at which the electrophoretic mobility vanished) was approximately 3.8.

**Figure 8:**
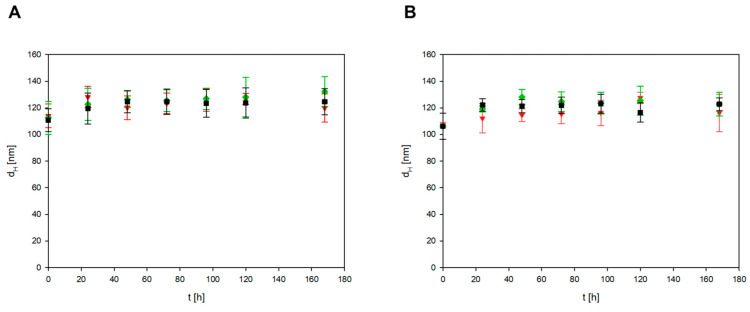
The dependence of the hydrodynamic diameter of the BN-14 (**A**) and BN-17 (**B**) particles on the incubation time. The points denote the experimental results obtained for DMEM supplemented with 10% FBS (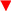), RPMI supplemented with 5% FBS (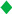), and RPMI supplemented with 10% FBS (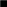). The BN bulk concentration was 200 mg L^−1^.

The stability of CBNs was also checked in three types of culture media (DMEM supplemented with 10% FBS, RPMI supplemented with 5% FBS, and RPMI supplemented with 10% FBS). Two types of CBN particles (BN-14 and BN-17) at a concentration of 200 mg/L were mixed with three types of culture media and then incubated at 37 °C for the specified time (7 days). As shown in Figure 8, the two types of CBN particles were stable for 175 h in all tested culture media because the hydrodynamic diameter did not increase significantly throughout the incubation period.

### 3.4. Effect of CBN Preparations on the Viability of Normal and Cancer Cells

**Figure 9:**
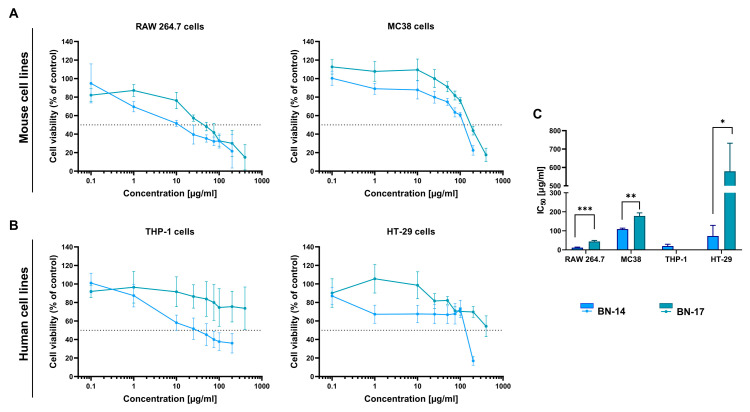
Effect of CBN preparations on cell proliferation. The viability of (**A**) mouse cells (RAW 264.7 and MC38) and (**B**) human cells (THP-1 and HT-29) after 72 h of exposure to CBN compounds (BN-14 and BN-17) was assessed using the MTT assay. The graphs represent the percentage of viable cells relative to that of control cells (control = 100%). (**C**) Concentrations of BN-14 and BN-17 causing 50% inhibition of cell proliferation (IC_50_) calculated for each cell line. BN-17 was not toxic to THP-1 cells; therefore, the IC_50_ value was not calculated. The results are expressed as the means ± SDs calculated for three independent experiments performed in triplicate. The differences between groups were calculated using multiple unpaired *t*-test (* *p* < 0.05; ** *p* < 0.01; *** *p* < 0.001).

The cytotoxicity of both synthesized CBN samples was assessed in mouse MC38 and RAW 264.7 cells (Figure 9A) and in human HT-29 and THP-1 cells (Figure 9B) using the MTT assay. After 72 h of exposure to the tested samples, the cellular sensitivity differed depending on the cell line type. In the case of MC38 and HT-29 cancer cells, CBN preparations at concentrations above 100 µg/mL decreased cell viability to less than 50%. However, as found in their comparison, BN-17 showed a significantly greater IC_50_ value than did BN-14. Thus, BN-17 was less toxic than BN-14 (Figure 9C).

The phagocytic cells represented herein by murine RAW 264.7 and human THP-1 cells were the most susceptible to the tested boron nitrides. For BN-14, the IC_50_ value was less than 20 µg/mL. Moreover, BN-17 had a greater inhibitory concentration (IC50) (<70 µg/mL) than did BN-14, and the differences were statistically significant. Similar to former cells, THP-1 monocytes showed greater sensitivity to BN-14. Nevertheless, it was not possible to calculate an IC_50_ value for BN-17 due to its low toxicity.

Considering the cytotoxicity results, BN-17 was less toxic than BN-14 to normal and cancer cells, possibly due to its better shape and size uniformity and low aggregation associated with good dispersion. Additionally, according to the physicochemical characteristics of the obtained boron nitride powders, the estimated presence of functional groups on the BN-17 surface seems greater than that on the BN-14 surface. Therefore, BN-17 was selected for further biological research.

### 3.5. Interaction of the FITC-Labeled Boron Nitride Compound with Normal and Cancer Cells

The ability of various mouse and human cell lines to bind BN-17 nanoparticles was examined using flow cytometry. For this purpose, the BN-17 compound was labeled with the fluorochrome FITC, and then the cells were incubated with this compound for 4 or 14 h. After this, flow cytometry was applied to analyze the fluorescence intensity and changes in the size and granularity of cells based on forward scatter (FSC) versus side scatter (SSC).

**Figure 10:**
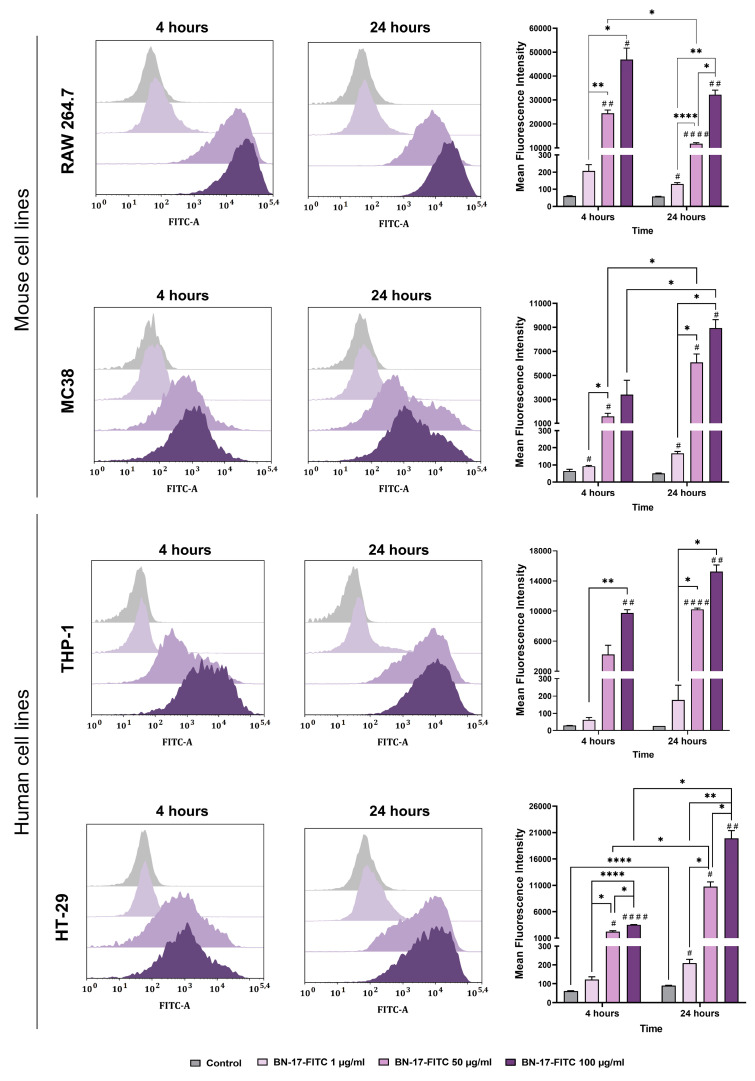
Interactions of FITC-labeled BN-17 with RAW 264.7 and MC38 mouse cells and with THP-1 and HT-29 human cells. Cells were exposed to FITC-labeled BN-17 (BN-17-FITC) at concentrations of 1, 50, and 100 µg/mL for 4 or 24 h. The mean fluorescence intensity (MFI) was analyzed by flow cytometry. Histograms showing changes in the MFI associated with the binding of BN-17-FITC to mouse and human cells. The bars represent the mean values ± SDs calculated for triplicate experiments. Differences between groups and time points were calculated using two-way ANOVA followed by Tukey’s multiple comparison post hoc test. The asterisks (*) presented in the graphs indicate statistically significant differences between the given groups; a hashtag (#) above a bar indicates statistically significant differences between the given group and the control group (*^/#^ *p* < 0.05, **^/##^ *p* < 0.01, and ****^/####^ *p* < 0.0001).

The ability of mouse cells to bind BN-17-FITC nanoparticles changed in a concentration- and time-dependent manner. The differences in the level of CBN binding were also related to the type of cell line (Figure 10). In the case of RAW 264.7 macrophages, an efficient interaction was noted 4 h after the addition of FITC-labeled BN-17. Extending the incubation time to 24 h resulted in a slight reduction in the MFI, which was statistically significant at a concentration of 50 µg/mL. In contrast, in the case of tumor MC38 cells, 24 h of exposure to the tested compound was needed to observe a higher MFI than the 4 h incubation time. Although MC38 cancer cells interacted with FITC-labeled BN-17, its binding was less effective than that of other cells. This phenomenon may be related to the increased uptake capacity of phagocytic cells, which are responsible for transporting stimuli and triggering an immune response, compared to cancer cells.

The binding of BN-17-FITC to human THP-1 and HT-29 cells was also analyzed. BN-17-FITC bound to cells in a concentration- and time-dependent manner, and the differences in the level of binding were mainly related to the type of cell line. In the case of the phagocytic THP-1 cells, a shorter period was sufficient to bind FITC-labeled BN-17, and prolonged incubation for 24 h did not significantly affect the further increase in the MFI. In contrast, the MFI after a 4 h incubation of HT-29 cells with FITC-labeled BN-17 was lower than that after 24 h. This again indicates that more efficient binding of the BN-17 compound to the cancer cell surface requires a longer incubation time.

**Figure 11:**
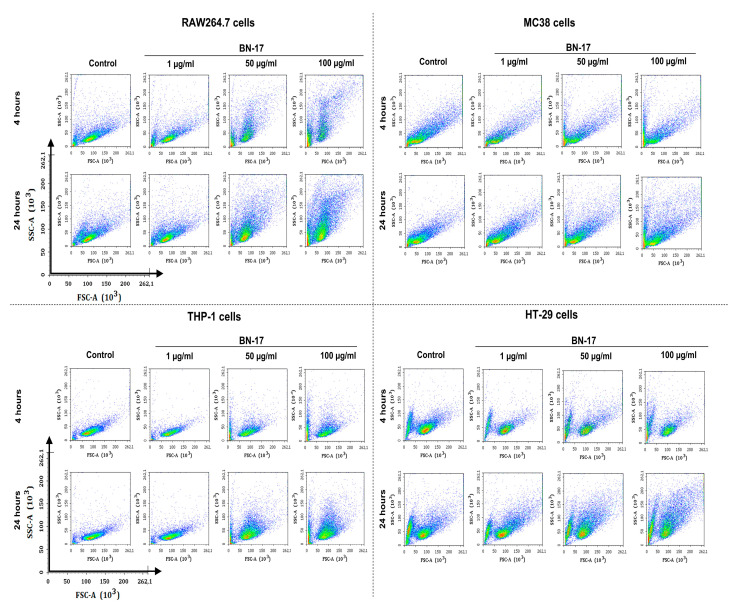
Flow cytometry density dot plots demonstrating changes in cell size and granularity based on forward scatter (FSC) versus side scatter (SSC) for murine RAW 264.7 and MC38 cells, as well as human THP-1 and HT-29 cells, after 4 and 24 h of exposure to the CBN preparation (BN-17-FITC) at concentrations of 1, 50, and 100 µg/mL compared to those of control untreated cells.

Analysis of the FSC vs. SSC density plots revealed that the increase in cell granularity was dependent on the BN-17-FITC concentration and incubation time (Figure 11). The greatest changes in granularity were observed in RAW 264.7 macrophages, while the smallest changes were observed in MC38 cells; this was consistent with the MFI results. To compare whether FITC labeling affects the growth of cell granulation over time, an additional analysis of the FSC vs. SSC density plots of unlabeled nanoparticles was performed on a flow cytometer (Figure S5). This analysis confirmed that FITC labeling did not significantly impact the interaction of the nanoparticles with the cells; the largest changes in cell granulation were still observed for the RAW 264.7 macrophages.

**Figure 12:**
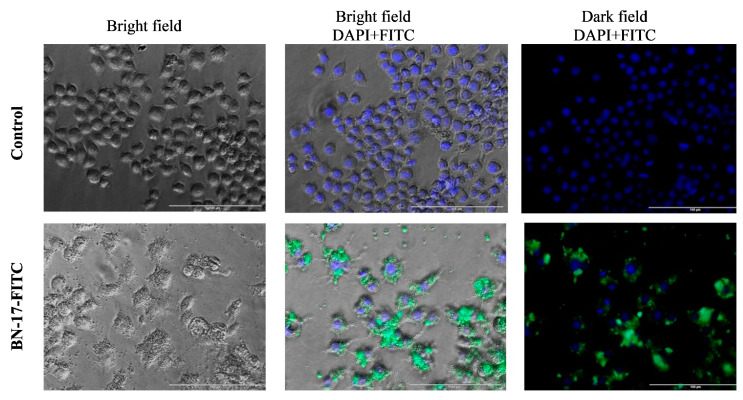
Fluorescence microscopy images showing the interaction of RAW 264.7 cells with FITC-labeled BN-17 compared to that of untreated control cells. RAW 264.7 cells were incubated with 100 µg/mL FITC-labeled BN-17 for 24 h. Cell nuclei were stained with DAPI. Images of the cells were taken under a bright field, and fluorescence was detected.

To confirm the effect of FITC-labeled BN-17 on the morphological changes in cells and thus confirm its ability to interact with the tested compound, visualization using a fluorescence microscope was performed (Figure 12). For this purpose, RAW 264.7 cells were selected because they had the greatest ability to interact with BN-17-FITC according to flow cytometry analysis. Visualization of RAW 264.7 cells treated with BN-17-FITC in bright fields revealed increased granularity compared with that of control cells. Moreover, fluorescence images confirmed the interaction of these cells with FITC-labeled BN-17.

The observed differences shed light on the fact that the degree of uptake of boron-rich molecules depends not only on the compound’s concentration in the growing cells but also on the type of cells and the time of their exposure to the agent.

## 4. Discussion

In summary, the results of our research on the synthesis of carbonated boron nitride in combustion wave propagating in mixtures of TAGAZ with NaBH_4_ or BH_3_NH_3_ demonstrated that this material could be used to efficiently produce nanosized CBN powders with particle structures and chemical compositions, which suggests its promising application in BNCT as well as in other biological and medical fields. As revealed by TGA-DTA measurements, the combustion products BN-14 and BN-17 contain 86.6 wt% and 82.8 wt% boron nitride with 7.9 at% and 13.5 at% boron atoms functionalized with hydroxyl groups, respectively. FTIR and XPS analyses confirmed the presence of covalently bonded hydroxyl groups and showed that the samples contained amino groups. XPS results also demonstrated the presence of boron-substituted graphite domains and carbon-substituted BN phases in the particles. In-depth characterization of the morphology, textural features, and chemical composition of the samples using HR-SEM and HRTEM methods indicated that the powders are composed of relatively loosely agglomerated hollow spheroidal nanoparticles whose walls are composed of several dozen concentric CBN layers with high tortuosity in their stacking sequence. Moreover, hydroxyl and amino groups probably separate the CBN layers because the interplanar spacing (002) was measured to be approximately 0.40 nm. However, the hexagonal in-plane order of the CBN particles still remains. The specific surface area of the tested samples is greater than 200 m^2^/g, which, together with the high degree of hydrophilic functionalization, allows the concentration of the aqueous BN-17 dispersion to reach 2 mg/mL.

Materials with poor suspension in physiological solutions are difficult to introduce into biological systems. We showed good dispersion of the obtained boron nitride preparations at a pH above 6 and a lack of visible aggregation in the culture media. These analyses performed using the DLS technique allowed the obtained nanoparticles to be qualified for further biological tests. Our observations are consistent with the literature, where it has been described that in the case of boron nitride nanotubes, the surface functionalization of these structures is of key importance for increasing the hydrophilicity of the surface and increasing the ability to evaluate the compounds received in in vitro and in vivo tests [45,46].

The assessment of the biological properties of CBN preparations allowed us to draw the following conclusions. The preparations significantly influenced cell viability, revealing that the toxicity of the BN-17 compound was lower than that of the BN-14 compound. Due to this observation, BN-17 nanoparticles were selected for further analysis. BN-17 can be bound and taken up by various cells, including both phagocytic and cancer cells. However, this could occur with different efficiencies related to their histological type and function in the body. The obtained nanoparticles had a hydrodynamic diameter of approximately 90 nm. According to the literature, such dimensions may cause toxicity in in vitro cell cultures. Mateti et al. demonstrated the relationship between the size of boron nitride particles and cytotoxicity, proving that nanometric particles show increasing toxicity with decreasing particle size in the preparation. Additionally, binding of the BN nanoparticles to the cell membrane may induce endocytosis, and the BN nanoparticles within the cell will generate ROS by triggering the oxidative stress signaling pathway. This can cause cell damage. The authors explained that a decrease in the diameter and thickness of the nanosheets led to an increase in the surface area and B content. The edges of small nanosheets have many unsaturated B atoms, which are chemically active. This phenomenon may cause toxicity to smaller particles [47].

On the other hand, the time and concentration dependence of BN-17 interactions with different cells indicates a wide range of possibilities for this boron-rich material. The shape and size uniformity and good dispersion favor the interaction of the received CBN particles with the biological environment. Additionally, the presence of functional groups inside and on the surface of BN-17 particles provides a broad spectrum of possible modifications, enabling selective boron delivery to the tumor tissue and specific targeting of various cell types by different membrane transport methods.

Studies of BN nanomaterials for biological applications are relatively new, and it is believed that they should be developed quickly, particularly concerning functionalized nano-BNs. The research presented in this paper fits into this trend. We propose the use of functionalized boron nitride nanoparticles obtained via the original procedure as promising boron carriers for boron neutron capture therapy. The physicochemical characteristics of the obtained preparations confirmed the possibility of their use in biological systems, and a preliminary assessment of the biological activity of the obtained nanoparticles showed that they do not interact equally with different cell types. In our opinion, further research on these compounds should lead to the biological functionalization of these nanoparticles to more effectively deliver boron to the tumor microenvironment and quantitatively assess the amount of accumulated boron in target cells.

## Data Availability

The raw data supporting the conclusions of this article will be made available by the authors on request.

## Supplementary Materials

The following supporting information can be downloaded at https://www.mdpi.com/article/10.3390/ma17102438/s1. Figure S1. XPS survey spectra and elemental compositions derived from the spectra. Figure S2. TEM images and elemental maps (B and N, C, O) for BN-14 (up) and BN-17 (down). Figure S3. EDX spectra of BN-14 (up) and BN-17 (down) samples. Figure S4. Nitrogen sorption-desorption isotherms for the BN-14 and BN-17 samples. Figure S5. Flow cytometry density dot plots demonstrating changes in cell size and granularity based on forward scatter (FSC) versus side scatter (SSC) for murine RAW 264.7 and MC38, as well as human THP-1 and HT-29 cells after 4 and 24-h exposure to CBN preparation (BN-17) at a concentration of 1, 50 and 100 µg/mL compared to control untreated cells.
