# Supplementary material for: Combustion Synthesis of Functionalized Carbonated Boron Nitride Nanoparticles and Their Potential Application in Boron Neutron Capture Therapy
Source: Materials (Basel). 2024 May 18;17(10):2438. doi: 10.3390/ma17102438 (PMC11122863; doi:10.3390/ma17102438)
Supplement: Supplementary file 1 [file materials-17-02438-s001.zip › materials-2959146-supplementary.pdf]

materials

Supplementary materials

Combustion Synthesis of Functionalized Carbonated Boron Nitride Nanoparticles and their Potential Application in Boron Neutron Capture Therapy

Stanisław Cudziło [1,*], Bożena Szermer-Olearnik [2,*], Sławomir Dyjak[1], Mateusz Gratzke [1], Kamil Sobczak [3], Anna Wróblewska [2], Agnieszka Szczygieł [2], Jagoda Mierzejewska [2], Katarzyna Węgierek-Ciura [2], Andrzej Rapak [2], Paulina Żeliszewska [4], Dawid Kozień [5], Zbigniew Pędzich [5] and Elżbieta Pajtasz-Piasecka [2]

[1] Faculty of Advanced Technologies and Chemistry, Military University of Technology, Warsaw, Poland;
[2] Hirszfeld Institute of Immunology and Experimental Therapy, Polish Academy of Sciences, Wrocław, Poland
[3] Faculty of Chemistry, Biological and Chemical Research Centre, University of Warsaw, Warsaw, Poland
[4] Jerzy Haber Institute of Catalysis and Surface Chemistry Polish Academy of Sciences, Krakow, Poland
[5] Department of Ceramics and Refractories, Faculty of Materials Science and Ceramics, AGH University of Krakow, Krakow, Poland
* Correspondence: S.C. (stanislaw.cudzilo@wat.edu.pl), B. S.O. (bozena.szermer-olearnik@hirszfeld.pl)

1. XPS characterization of BN-14 and BN-17

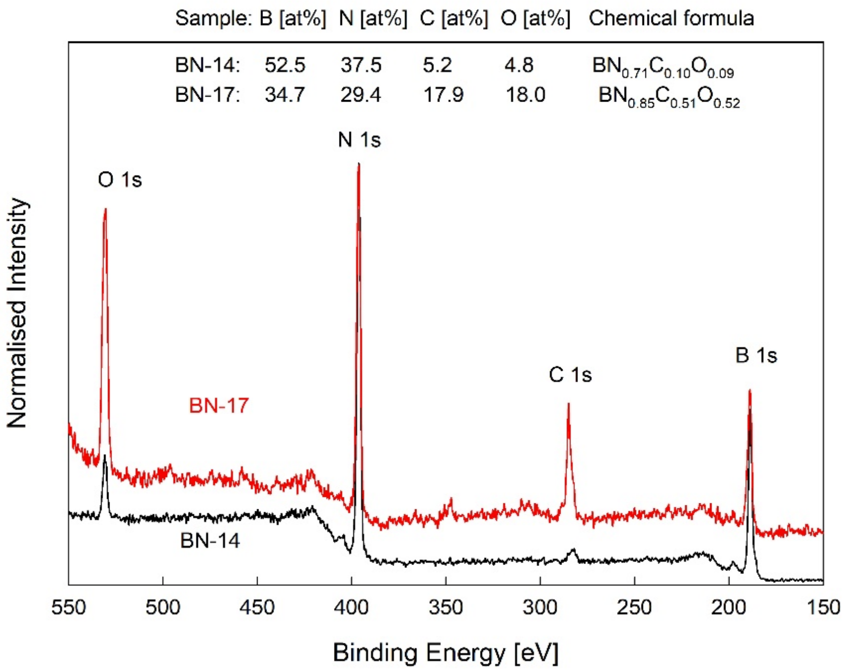

Figure S1. XPS survey spectra and elemental compositions derived from the spectra.

2. HAADF imaging of BN-14 and BN-17

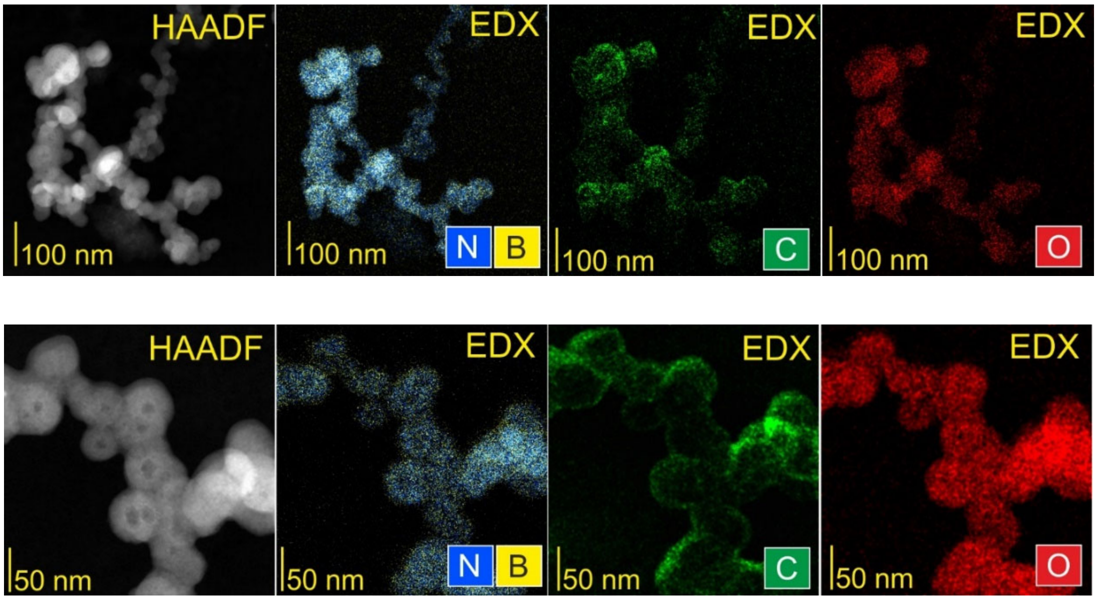

Figure S2. TEM images and elemental maps (B and N, C, O) for BN-14 (up) and BN-17 (down).

3. EDX analysis of BN-14 and BN-17

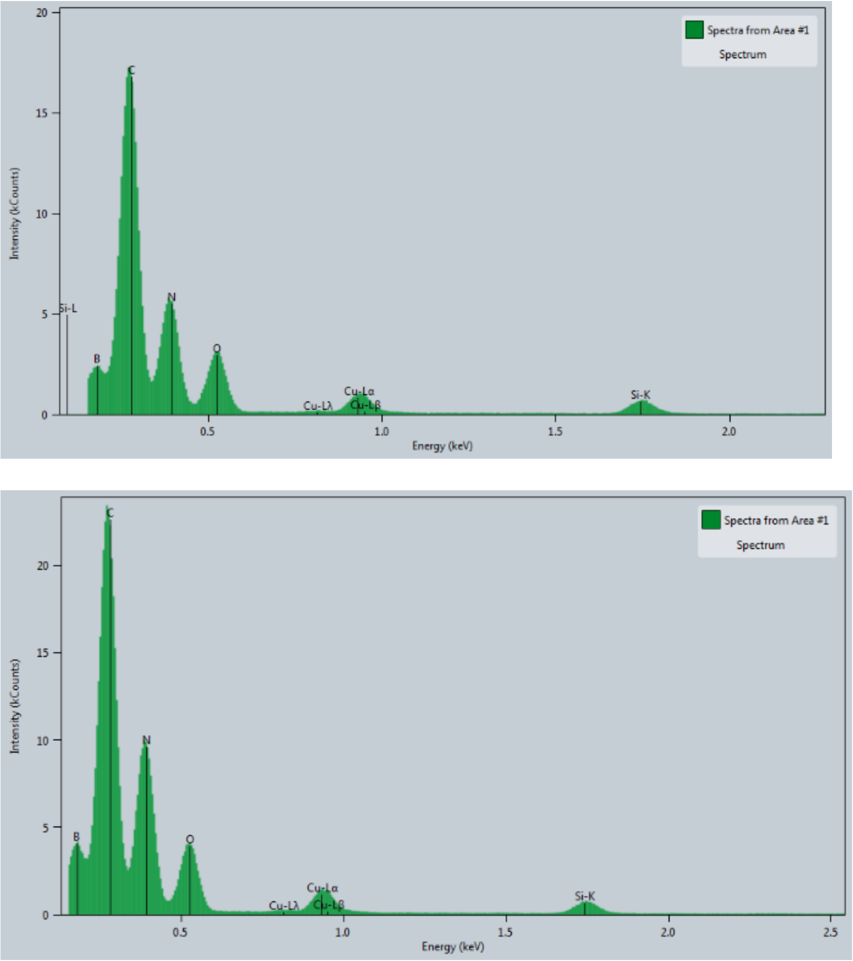

Figure S3. EDX spectra of BN-14 (up) and BN-17 (down) samples.

4. Low temperature nitrogen sorption analysis

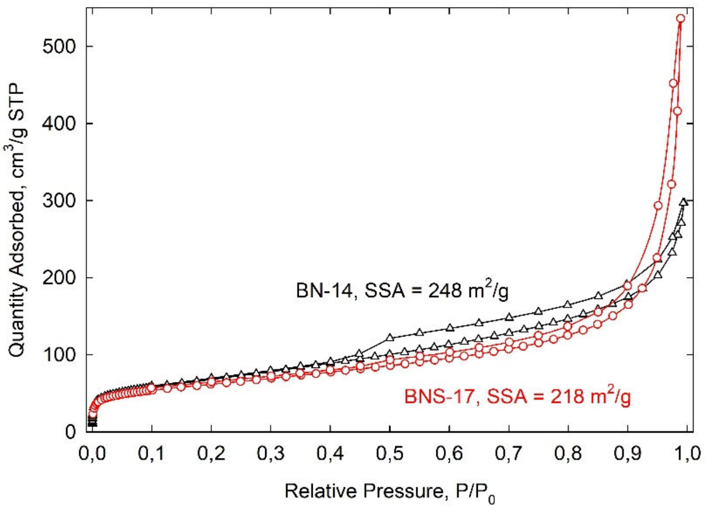

Figure S4. Nitrogen sorption-desorption isotherms for the BN-14 and BN-17 samples.

5. Flow cytometry analysis of cell size and granularity

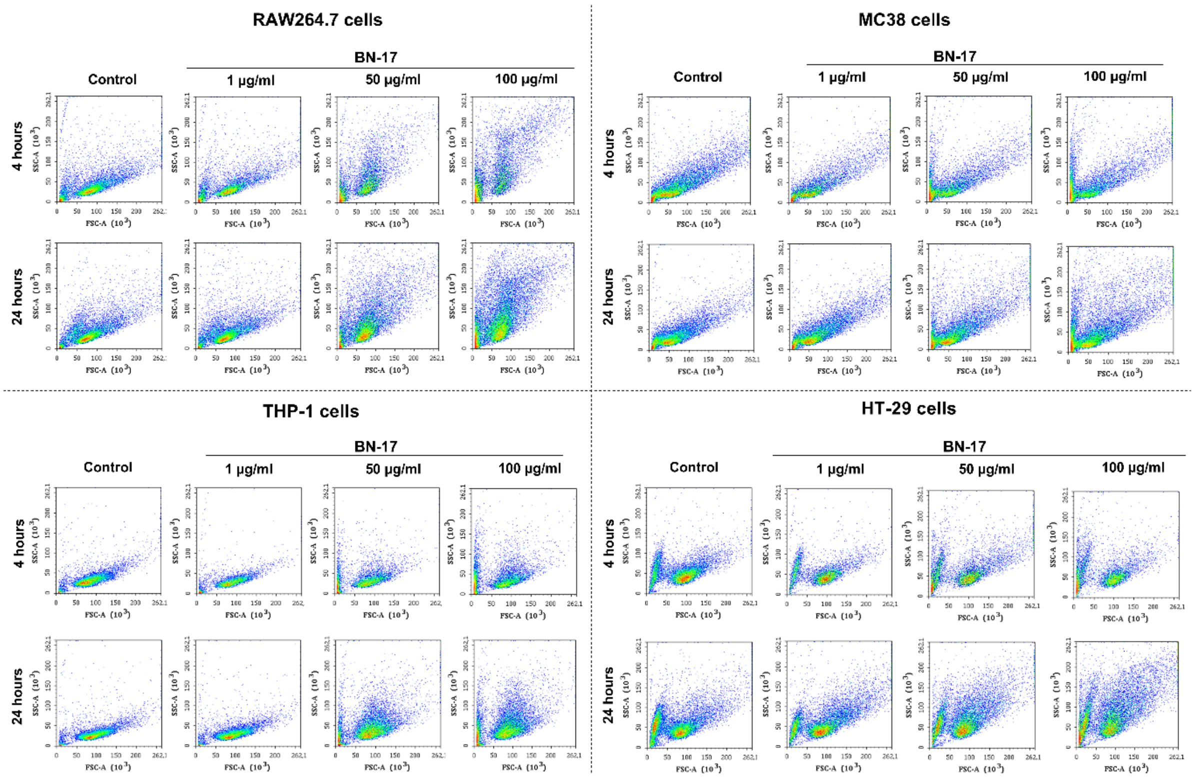

Figure S5. Flow cytometry density dot plots demonstrating changes in cell size and granularity based on forward scatter (FSC) versus side scatter (SSC) for murine RAW 264.7 and MC38, as well as human THP-1 and HT-29 cells after 4 and 24-hour exposure to CBN preparation (BN-17) at a concentration of 1, 50 and 100 µg/ml compared to control untreated cells.
